# Cyst stem cell lineage GAL4 transgenes are robustly expressed in hub cells of the *Drosophila* testis stem cell niche

**DOI:** 10.17912/micropub.biology.002106

**Published:** 2026-04-17

**Authors:** Jennifer Viveiros, Erika Matunis

**Affiliations:** 1 Cell Biology, Johns Hopkins Medicine, Baltimore, MD, US

## Abstract

Manipulating gene expression in the different cell types of the *Drosophila melanogaster* testis stem cell niche is critical to understanding its biology. Here, we tested the specificity of commonly used somatic cyst stem cell (CySC) lineage GAL4 drivers by assessing their abilities to promote loss of hub cell quiescence. We found that conditional reduction of the cell cycle inhibitor Retinoblastoma (Rbf) using *c587-GAL4* , *tj-GAL4* , *eyaA3-GAL4* , or *zfh1-GAL4* is sufficient to drive quiescent hub cells into mitosis, with increased penetrance at higher temperature due to higher GAL4 activity. Concomitant inhibition of GAL4 expression in hub cells via *hh-GAL80* eliminates hub cell proliferation, supporting that hub cells autonomously require Rbf to maintain quiescence. Thus, the activity of GAL4 drivers commonly used to manipulate the CySC lineage also functionally affect hub cells.

**Figure 1. Cyst stem cell lineage GAL4 drivers can robustly drive UAS-transgene expression in hub cells:** ( **A** ) Illustration of the testis apex containing niche (hub) cells (green) which are surrounded by and maintain Germline Stem Cells (GSCs, purple) and Cyst Stem Cells (CySCs, blue). As stem cells divide, their progeny move away from the hub and differentiate. GSCs give rise to gonialblasts (GBs) that divide to produce spermatogonia (SG) which undergo three additional rounds of transit amplification and ultimately produce sperm. CySCs divide and produce daughter cyst cells (CCs) that differentiate as they support adjacent germ cells. ( **B – G** ) Representative single confocal sections through testis apices from flies carrying the cyst cell driver, *c587-GAL4;; tub-GAL80 ^TS^* , controlling expression of (B) *UAS-GFP RNAi* (control), (C,F) *UAS-Rbf RNAi* , or (D – E,G) *UAS-RFP* in the (B – E) absence or (F – G) presence of *hh-GAL80* . Testes were stained for hub cell membranes (Fasciclin3 (FasIII), green), nuclei (DAPI, blue), and (B – C,F) mitotic cells (phospho-histone H3 (PH3), green) or (D – E,G) RFP (DsRed, magenta, insets). Duration and temperature of GAL4 induction indicated on images. Scale bars, 20 μm. Hubs outlined (dashed yellow lines).

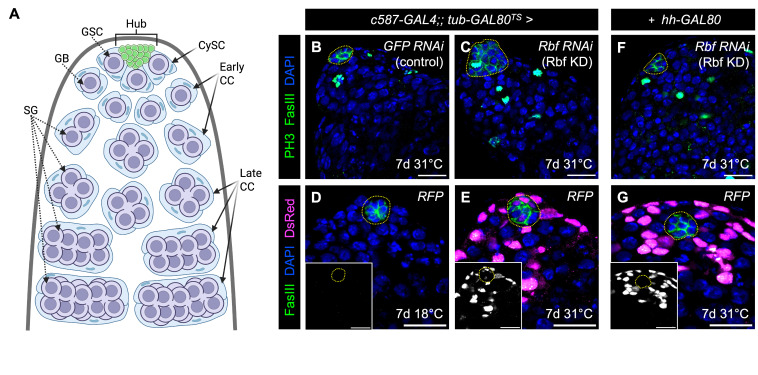



## Description

Among its numerous attributes, the ability to regulate gene expression in both a temporal and spatial manner makes *Drosophila melanogaster* one of the leading model organisms. Additionally, the public availability of numerous tissue-specific GAL4 transgenes allows for even greater genetic control (Brand & Perrimon, 1993; McGuire, Roman, et al., 2004; Jenkins et al., 2022; Zirin et al., 2024). &nbsp;

&nbsp;

Studies using the *Drosophila* testis stem cell niche have relied upon the specificity of GAL4 transgenes to spatially restrict UAS transgene expression to specific groups of cells. The adult testis niche is located within the apical end of the testis, a coiled, blind-ended tubule. The niche houses three cell types: the germline stem cells (GSCs), the cyst stem cells (CySCs), and the niche cells, which are referred to as the hub. The GSCs are the sperm-producing cells of the testis and the somatic CySCs and daughter cyst cells (CCs) act to support GSCs and their daughters. The maintenance of the stem cell populations relies on the hub, a small cluster of tightly packed, quiescent cells, anchored to the testis’ apical end. (reviewed in (Greenspan et al., 2015; Siddall & Hime, 2017)).

&nbsp;

To accurately interrogate stem cell niche biology there must be GAL4 transgenes specific to each cell type. The nature of the germline makes specificity achievable with transgenes like *nanos-GAL4* (Van Doren et al., 1998) and *bam-GAL4* (Chen & McKearin, 2003). By contrast, the hub cells, CySCs, and CCs are all somatic cells, descending from the same population of mesodermal cells in the embryo (Gehring et al., 1976; Lawrence & Johnston, 1986; Szabad & Nöthiger, 1992). Previous work has relied on the *E132-GAL4* (also known as *upd1-GAL4* ) to control UAS transgene expression in hub cells (Kawase et al., 2004). For expression in CySCs and/or their daughters, several GAL4 transgenes have been frequently used, with differing degrees of expression patterns ranging from broad ( *c587-GAL4* (Kawase et al., 2004) and *tj-GAL4* (Li et al., 2003; Tanentzapf et al., 2007)) to more restricted (CySCs and early CCs: *zfh1-GAL4* (Albert et al., 2018), *fng-Gal4* (DiNardo et al., 2011), *c833-GAL4* (Papagiannouli & Mechler, 2009), and *ptc-GAL4* &nbsp;(Schulz et al., 2004); early and late CCs: *spict-GAL4* (Chiang et al., 2017) and *eyaA3-GAL4* (Leatherman & DiNardo, 2008)). However, these expression patterns can vary; when flies are incubated at higher temperatures the range of *eyaA3-GAL4* expression has been shown to extend into CySCs (Ma et al., 2014) and Zfh1 protein expression increases in hub cells (Hétié et al., 2014). And of most significance, studies have noted that some of these GAL4 drivers ( *c833-GAL4* and *tj-GAL4* in particular) are also expressed in hub cells, often demonstrated through fluorescent reporter expression (Papagiannouli & Mechler, 2009; Joti et al., 2011; Fairchild et al., 2016). However, the extent of CySC lineage driver expression in hub cells has not been well assayed.

&nbsp;

To better understand the degree of CySC lineage GAL4 expression in the adult hub, we surveyed expression of four well-used transgenes: *c587-GAL4* , *tj-GAL4* , *eyaA3-GAL4* , and *zfh1-GAL4* . We combined each GAL4 driver with a ubiquitously expressed temperature sensitive (TS) GAL4 inhibitor, *tub-GAL80 ^TS^* , to suppress UAS construct expression during development (McGuire, Mao, et al., 2004). After eclosion, adult flies were transferred from 18°C to 29°C or 31°C, to inactivate GAL80 ^TS^ and trigger GAL4 activation of UAS transgenes. Due to the subjective nature of fluorescent reporter expression, we sought a reliable and sensitive read-out of GAL4 activity. We have previously demonstrated that hub cells are kept quiescent by the cell cycle inhibitor Retinoblastoma-family protein (Rbf) and that when hub cells lose Rbf ( *E132-GAL4;; tub-GAL80 ^TS^ / UAS-Rbf RNAi* ), they enter mitosis and proliferate. Therefore, we hypothesized that if CySC lineage GAL4 drivers are active in hub cells, we would see an increase in the frequency of loss of hub cell quiescence upon GAL4-mediated Rbf reduction. As previously described, mitotic hub cells were identified by immunostaining testes for the mitotic marker phospho-histone H3 (PH3); testes with at least one PH3-positive hub cell were scored as positives (Greenspan & Matunis, 2018; Greenspan et al., 2022). As expected, expression of the control RNAi transgene ( *UAS-GFP RNAi* ) did not produce mitotic hub cells in any genotype under any condition ( **Fig. 1B, Table 1** ). By contrast, Rbf reduction by any one of the four CySC lineage GAL4 genotypes resulted in significantly higher frequencies of PH3-positive hub cell(s) following incubation at 31°C compared to control RNAi ( **Fig. 1C, Table 1** ). Surprisingly, the frequencies of testes with PH3-positive hub cell(s) were similar to that of Rbf reduction in hub cells alone using the hub specific *E132-GAL4* driver following 7 days at 31°C (29% of testes (Greenspan et al., 2022)). However, incubation of GAL4-mediated Rbf knockdown flies at 29°C produced fewer or no testes with PH3-positive hub cells(s) ( **Table 1** ). This finding supports previous work that CySC lineage GAL4 driver expression is dependent on temperature and extends this expression pattern to include hub cells (Ma et al., 2014; Greenspan & Matunis, 2018). Altogether, these results demonstrate that GAL4 transgenes typically used for their expression in CySCs and their daughters can drive robustly in hub cells as well, especially when subjected to higher temperatures.

&nbsp;

Studies have started to consider the expression of CySC lineage GAL4 drivers in the hub (Herrera et al., 2021; ; Sainz de la Maza et al., 2025; Grace et al., in prep). As such, a hub-specific GAL80 controlled by the endogenous hedgehog locus ( *hh-GAL80* ) was created to inactivate GAL4 expression in the hub (Herrera et al., 2021). In a similar approach, we introduced the *hh-GAL80* transgene into the CySC lineage GAL4 drivers to demonstrate that the occurrence of PH3-positive hub cells following Rbf knockdown was indeed due to expression of GAL4 protein in hub cells. We first sought to confirm that the *hh-GAL80* does suppress GAL4 expression in adult hub cells. First, we assessed testes from flies expressing *UAS-RFP* under the control of one of the CySC lineage GAL4 drivers ( *c587-GAL4;; tub-GAL80 ^TS^* ) in the absence of *hh-GAL80* . As anticipated, we did not observe RFP in testes of flies kept at 18°C ( **Fig. 1D** ). However, after incubation at 31°C, we observed robust expression of RFP in the cyst lineage as well as some, but not all, hub cells ( **Fig. 1E** ). When we added *hh-GAL80* to this genetic background ( *c587-GAL4; tub-GAL80 ^TS^ ; hh-GAL80* ), RFP expression in the hub was eliminated, confirming the specificity and efficacy of the GAL80 transgene ( **Fig. 1G** ). Further, when we assayed loss of hub quiescence, the addition of *hh-GAL80* resulted in significant loss of PH3-positive hub cell(s) in testes of all four CySC lineage GAL4 drivers ( **Fig. 1F, Table 1** ). This confirms that indeed the loss of hub cell quiescence observed following Rbf reduction by the CySC lineage GAL4 drivers was due to GAL4 activity within hub cells, as opposed to a non-cell autonomous mechanism caused by Rbf reduction in surrounding CySCs.

**Table 1. Concomitant inhibition of cyst stem cell lineage GAL4-mediated Rbf knockdown in hub cells eliminates loss of hub cell quiescence.**

**Table:** 

&nbsp;		&nbsp;	**% Testes with PH3-positive hub cell(s)**		
**CySC Lineage GAL4 Driver**	**Presence of** ***hh-GAL80* transgene **	**UAS line**	**7-10 days at 18°C**	**7-10 days at 29°C**	**7-10 days at 31°C**
* c587-GAL4;; tub-GAL80 ^TS^*	-	*UAS-GFP RNAi*	0%&nbsp;&nbsp; (0/99)	0%&nbsp;&nbsp; (0/28)	0%&nbsp; (0/123)
	-	*UAS-Rbf RNAi* (III)	0%&nbsp;&nbsp; (0/97)	5%&nbsp;&nbsp; (4/74) ^§,ns^	42%&nbsp; (83/199) ^§,****^
	+		0%&nbsp;&nbsp; (0/35)	--	0%&nbsp; (0/148) ^a,****^
* tj-GAL4; tub-GAL80 ^TS^*	-	*UAS-GFP RNAi*	0%&nbsp;&nbsp; (0/70)	0%&nbsp;&nbsp; (0/123)	0%&nbsp; (0/179)
	-	*UAS-Rbf RNAi* (III)	0%&nbsp;&nbsp; (0/98)	0%&nbsp;&nbsp; (0/105) ^§,ns^	37%&nbsp; (122/333) ^§,****^
	+		0%&nbsp;&nbsp; (0/27)	--	0%&nbsp; (0/109) ^a,****^
* eyaA3-GAL4; tub-GAL80 ^TS^*	-	*UAS-GFP RNAi*	0%&nbsp;&nbsp; (0/80)	0%&nbsp;&nbsp; (0/45)	0%&nbsp; (0/104)
	-	*UAS-Rbf RNAi* (III)	0%&nbsp;&nbsp; (0/112)	3%&nbsp;&nbsp; (3/90) ^§,ns^	41%&nbsp; (115/278) ^§,****^
	+		0%&nbsp;&nbsp; (0/37)	--	0%&nbsp; (0/78) ^a,****^
* tub-GAL80 ^TS^ ; zfh1-GAL4* &nbsp;	-	*UAS-GFP RNAi*	0%&nbsp;&nbsp; (0/25)	0%&nbsp;&nbsp; (0/59)	0%&nbsp; (0/35)
	-	*UAS-Rbf RNAi* (II)	0%&nbsp;&nbsp; (0/39)	12%&nbsp;&nbsp; (3/25) ^§,*^	18%&nbsp; (12/66) ^§,**^
	+		0%&nbsp;&nbsp; (0/10)	--	1%&nbsp; (1/108) ^a,****^

Analyses using Fisher's&nbsp;exact&nbsp;test. (§) denotes comparison to respective GFP control. (a) denotes comparison between expression of *UAS-Rbf RNAi* with or without the *hh-GAL80* transgene.

&nbsp;

This work demonstrates that GAL4 transgenes commonly used to drive gene expression in CySCs and their daughters are also active in hub cells. Further, this expression is to such a degree that it can regulate UAS transgenes to generate pathological phenotypes, including the proliferation of normally quiescent cells. Future work using cyst lineage GAL4 drivers should consider hub expression and verify the specificity with transgenes like *hh-GAL80* .

## Methods

**Fly husbandry and stocks**

Flies were raised on a standard yeast/cornmeal/molasses medium supplemented with dry yeast as previously described (Greenspan et al., 2022). Crosses were set and matured at 18°C. Male flies were used for all experiments and subjected to conditions as noted within the text, figure legend, and methods. See Reagents Table for a list of strains used in this study. The *hh-GAL80* transgene was used in combination with GAL4 transgenes to inhibit GAL4 expression in hub cells (Herrera et al., 2021).

&nbsp;

**Temperature-based transgene induction**

As previously described (McGuire, Mao, et al., 2004), flies containing UAS and Gal4 constructs together with *tub-GAL80 ^TS^* transgenes were raised at the permissive temperature (18°C). Following eclosion, approximately 25 young male progeny (0-4 days old) were transferred into new vials and either kept at 18°C or incubated at restrictive temperatures (29°C or 31°C) for 7-10 days.

&nbsp;

**Testis dissection and immunofluorescence**

Testis dissection and immunofluorescence were performed as previously described (Matunis et al., 1997). All steps were performed at room temperature and on a nutating platform unless otherwise noted. CO _2_ -anesthetized male flies were dissected in 1X Ringer’s solution. Testes with attached cuticle were transferred to fixative (4% paraformaldehyde in 1X PBS with 0.1% Triton X-100 (1X PBX)) for 20 min. After fixation, testes were rinsed two times followed by a 30 min wash in 1X PBX and then incubated in block solution (3% BSA and 0.02% NaN _3_ in 1X PBX) supplemented with 2% normal goat serum (Millipore/Sigma, G9023) for one hour at room temperature or overnight at 4°C. Testes were then incubated in primary antibodies diluted in block overnight at 4°C, rinsed as above, incubated for an hour in PBX, then incubated in secondary antibodies and 4,6-diamidino-2-phenylindole (DAPI; 1μg/mL, Millipore/Sigma, 10236276001) diluted in block for 1.5 hrs at room temperature or overnight at 4°C. Testes were rinsed and washed for an hour in PBX with final rinses and a 10 min wash in 1X PBS before transfer to Vectashield (Vector Laboratories, H-1000). Samples were stored at -20°C prior to imaging. Primary antibodies used were against Fasciclin3 (FasIII; 1:50, DSHB, 7610) and phosho-histone H3 (PH3; 1:500, Cell Signaling, 9701), and DsRed (1:5,000, Takara Bio, 632496). Secondary antibodies used were Goat anti-Mouse IgG (H+L) Cross-Adsorbed Secondary Antibody, Alexa Fluor™ 488 (1:200, Thermo Fisher Scientific, A-11073), Goat anti-Rabbit IgG (H+L) Cross-Adsorbed Secondary Antibody, Alexa Fluor™ 568 (1:100, Thermo Fisher Scientific, A-11011), and Goat anti-Mouse IgG (H+L) Highly Cross-Adsorbed Secondary Antibody, Alexa Fluor™ 647 (1:66, Thermo Fisher Scientific, A-21236).

&nbsp;

**Microscopy and image analysis**

Testes were mounted on standard slides under #1.5 thickness coverslips. Images were obtained using a Zeiss LSM 800 microscope equipped with a 63x oil immersion objective 405 nm, 488 nm, 561 nm, and 640 nm diode lasers with digital zoom and GaAsP and Airyscan detectors. Images were acquired using Zen software with Z-stacks acquired using a 0.5 μm step, followed by processing using Zen or FIJI. Brightness for individual channels from single confocal slices was enhanced using Zen or FIJI, and then the channels were overlaid to form a merged image. Single slices are shown in this paper.

&nbsp;

**Quantification of dividing hub cells**

To identify hub cells in mitosis, testes were immunostained with an antibody to the mitotic marker PH3 as previously described&nbsp; (Greenspan & Matunis, 2018; Greenspan et al., 2022). Testes with at least one hub cell (as identified by the membrane marker FasIII), that was PH3-positive were scored as positive for dividing hub cells.

**Statistical analysis**

All statistical analysis was performed using GraphPad Prism 11. For all statistical analysis, significant pairwise comparisons are shown as follows: ns = not significant, *p value < 0.05, **p value < 0.01, ***p value < 0.001, and ***p value < 0.0001.

## Reagents

**Table:** 

**Strain**	**Genotype**	**Source or Reference**
*c587-GAL4*	P{w[+mW.hs]=GawB}C587, w[*]	BDSC_67747
*tj-GAL4*	*y w; P{GawB}NP1624/CyO, UAS-LacZ*	DGRC 104055
*zfh1-GAL4*	*zfh1::T2A::GAL4/TM6B, Tb*	Gift of E. Bach (Albert et al., 2018)
*eyaA3-GAL4*	*eyaA3-GAL4*	S. DiNardo (Leatherman and DiNardo, 2008)
* tub-GAL80 ^TS^*	w[*]; P{w[+mC]=tubP-GAL80[ts]}10; TM2/TM6B, Tb[1]	BDSC_7108
* tub-GAL80 ^TS^*	w[*]; P{w[+mC]=tubP-GAL80[ts]}2/TM2	BDSC_7017
*UAS-Rbf RNAi (III)*	y[1] sc[*] v[1] sev[21]; P{y[+t7.7] v[+t1.8]=TRiP.HMS03004}attP2/TM3, Sb[1]	BDSC_36744
*UAS-Rbf RNAi (II)*	y[1] sc[*] v[1] sev[21]; P{y[+t7.7] v[+t1.8]=TRiP.GL01293}attP40	BDSC_41863
*UAS-GFP RNAi*	*w[1118]; P{w[+mC]=UAS-GFP.RNAi.R}142*	BDSC_9330
*UAS-RFP*	*w[1118]; P{w[+mC]=UAS-RedStinger}6*	BDSC_8547
*hh-GAL80*	* GAL80 ^hh-MI10526-T3XG80.0^*	E. Bach (Herrera, et al., 2021)
